# Gambling Near-Misses Enhance Motivation to Gamble and Recruit Win-Related Brain Circuitry

**DOI:** 10.1016/j.neuron.2008.12.031

**Published:** 2009-02-12

**Authors:** Luke Clark, Andrew J. Lawrence, Frances Astley-Jones, Nicola Gray

**Affiliations:** 1 Behavioural and Clinical Neuroscience Institute, Department of Experimental Psychology, University of Cambridge, CB2 3EB Cambridge, UK

**Keywords:** SYSNEURO

## Abstract

“Near-miss” events, where unsuccessful outcomes are proximal to the jackpot, increase gambling propensity and may be associated with the addictiveness of gambling, but little is known about the neurocognitive mechanisms that underlie their potency. Using a simplified slot machine task, we measured behavioral and neural responses to gambling outcomes. Compared to “full-misses,” near-misses were experienced as less pleasant, but increased desire to play. This effect was restricted to trials where the subject had personal control over arranging their gamble. Near-miss outcomes recruited striatal and insula circuitry that also responded to monetary wins; in addition, near-miss-related activity in the rostral anterior cingulate cortex varied as a function of personal control. Insula activity to near-misses correlated with self-report ratings as well as a questionnaire measure of gambling propensity. These data indicate that near-misses invigorate gambling through the anomalous recruitment of reward circuitry, despite the objective lack of monetary reinforcement on these trials.

## Introduction

Gambling is a prevalent and culturally ubiquitous form of entertainment that becomes dysfunctional in a small but significant minority (1%–5%), in whom it resembles a substance addiction in several core respects (Potenza, 2006; Shaffer et al., 2004). The popularity of gambling might seem surprising given the widespread acceptance among those who gamble that “the house always wins.” This refers to the notion that the expected value of gambling is negative, such that the player will lose money over time. In order to identify the etiological processes in problem (or “pathological”) gambling, it is necessary to understand the allure of gambling within wider society. Cognitive formulations of gambling (e.g., Langer, 1975; Wagenaar, 1988; Walker, 1992) propose that certain characteristics of gambling games foster an exaggerated confidence in one's chances of winning. Thus, even though the winning outcomes are determined largely or purely by chance, the gambler develops an “illusion of control” such that he believes he can master the game and recoup his past losses.

In this study, we focus on two common characteristics of gambling games that can be modeled in the laboratory and are known to promote gambling tendencies: the impact of near-misses and the influence of personal control. Near-misses occur when an unsuccessful outcome is proximal to the designated win, such as when a chosen horse finishes in second place or when two cherries are displayed on the slot machine payline. Their significance to the gambler has long been recognized (e.g., Reid, 1986), to the extent that the misappropriation of slot machine near-misses has been the focus of legal cases (Harrigan, 2008). Studies manipulating the frequency of near-misses have shown effects on gambling persistence (Cote et al., 2003; Kassinove and Schare, 2001), which follow an inverted-U shaped function that is maximal around 30% (Kassinove and Schare, 2001). As a consequence of near-misses, the gambler may feel that he is “not constantly losing but constantly nearly winning” (Griffiths, 1991). These accounts of near-misses emphasize their positive, hedonic value, such that we predicted recruitment of brain reward circuitry during near-miss outcomes, despite the objective lack of monetary reinforcement on these trials.

The second factor that was modeled in our task was personal control, which refers to the gambler's level of involvement in arranging their gamble. On games of pure chance like the lottery, craps, and roulette, gamblers have an equal chance of winning regardless of whether they, or another agent, places the gamble. However, it is repeatedly observed that gamblers have inflated confidence (indicated by wager size, for example) when given the opportunity to choose their lottery ticket or throw the dice or roulette ball themselves, compared to conditions where the action is performed by another (Davis et al., 2000; Ladouceur and Mayrand, 1987; Langer, 1975). Craps players also use harder throws when trying to roll high numbers (Henslin, 1967). The presence of personal control may be a core factor in causing the gambler to mistake a game of chance for a game with some skill component, which is effectively controllable. Instrumentality (i.e., the requirement of an active response) has been shown to influence the neural correlates of feedback processing in recent brain imaging (O'Doherty et al., 2004; Tricomi et al., 2004; Walton et al., 2004) and electroencephalography (Yeung et al., 2005) studies with monetary reward tasks. We reasoned that if near-miss outcomes promoted gambling behavior by fostering an illusion of control, their efficacy would be greater on trials where the gambler had personal control, compared to trials where the gamble was arranged by the computer.

The aims of the present study were threefold: first, to devise a task to elicit near-miss and control phenomena in the laboratory, as measured by self-report ratings administered on a trial-by-trial basis. Second, we explored the neural mechanisms underlying these cognitive distortions, using functional magnetic resonance imaging (fMRI). We were specifically interested in a neural system comprising the ventral striatum and medial frontal cortex, which previous research has robustly implicated in processing unexpected monetary wins (Breiter et al., 2001; Delgado et al., 2000; Elliott et al., 2000; Knutson et al., 2003), as well as primary rewards (e.g., fruit juice) (Berns et al., 2001; McClure et al., 2003), social rewards (acquisition of good reputation; Izuma et al., 2008), and drugs of abuse (Breiter et al., 1997; Gilman et al., 2008; Stein et al., 1998). Third, we examined the associations between the level of activation in this circuitry during gambling and a trait measure of gambling propensity, the gambling-related cognitions scale (GRCS; Raylu and Oei, 2004). This self-report questionnaire assesses the susceptibility to common gambling distortions like predictive control (the belief that one can predict when a win is due) and interpretive bias (reframing gambling outcomes to encourage further play; see Table S1 available online). In contrast to clinically oriented gambling scales such as the South Oaks Gambling Screen (SOGS; Lesieur and Blume, 1987), GRCS scores are well-distributed within samples of non-problem gamblers (Raylu and Oei, 2004).

**Figure 1:**
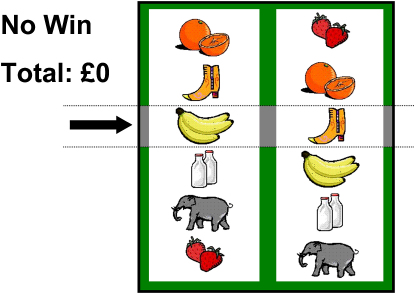
Screen Display for the Slot Machine Task The slot machine task displayed two reels, a payline and a box displaying current earnings. Both reels displayed the same six icons. Each trial commenced with a *selection phase* (duration 5 s), where either the participant or the computer selected the “play icon” on the left-hand reel. The right-hand reel then spun for a variable *anticipation phase* (duration 2.8–6 s), decelerating to a standstill. In the *outcome phase* (duration 4 s), if the play icon on the right-hand reel stopped in the payline (i.e., was aligned with the chosen play icon on the left-hand reel), the participant won £0.50. Other outcomes yielded no win; it was not possible for the participant to lose money. Trials where the right-hand reel reached a standstill one position from the payline (either above or below) were classified as near-misses, and trials where the right-hand reel stopped more than one position from the payline were classified as full-misses. During the selection phase, participants performed a pseudorandom sequence of participant-chosen (white background) and computer-chosen (black background) trials. On participant-chosen trials, the subject was able to rotate the left-hand reel and select a play icon by moving it around to the payline. On computer-chosen trials, the computer would rotate the left-hand reel until one play icon was highlighted on the payline; the subject was required to confirm selection with a button press to ensure adequate attention.

We developed a gambling task (see Figure 1) resembling a slot machine with two reels, each displaying six icons and a payline. On each trial, the participant could either win (£0.50) or not win. Personal control was manipulated by having the participant choose the play icon on some trials, and the computer chose the play icon on other trials. Following selection of the play icon on the left reel, the right reel was spun for an anticipatory period and slowed to a standstill on one icon. Wins occurred on 1/6 of trials, when the right reel stopped with the play icon in the payline. Near-misses occurred on 2/6 of trials, when the play icon stopped one position from the payline. These frequencies are comparable to real-world slot machines (Griffiths, 1993), and previous work has shown optimal gambling persistence at a 30% frequency of near-misses compared to 15% and 45% frequencies (Kassinove and Schare, 2001). All other outcomes (3/6 of trials) where the play icon stopped more than one position from the payline were designated “full-misses.”

## Results

### Experiment I: Behavioral Effects of Near-Misses and Personal Control

**Figure 2:**
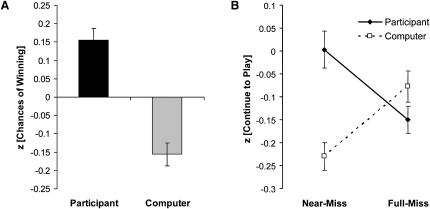
Subjective Ratings on the Slot Machine Task Ratings for each subject were standardized based on the individual's mean and standard deviation for that rating, given the variability in anchoring across subjects. (A) Ratings of “How do you rate your chances of winning?” were significantly higher on participant-chosen trials compared to computer-chosen trials (t_39_ = 5.09, p < 0.001). (B) Ratings of “How much do you want to continue to play the game?” on near-miss and full-miss outcomes. The “near-miss effect” (increased desire to play after near-misses) was restricted to trials where the subject had personal control over the gamble; near-misses selected by the computer significantly reduced desire to play. Error bars indicate standard error of the mean.

In a behavioral experiment in healthy volunteers (n = 40), three sets of self-report ratings were taken on each trial. Immediately after the selection phase, participants were asked “How do you rate your chances of winning?” After the outcome phase, two further ratings were taken: “How pleased are you with the result?” and “How much do you want to continue to play the game?” Each subjects' ratings were z transformed to their own mean and standard deviation for statistical analysis. Personal control over the gamble was associated with significantly higher ratings of “chances of winning,” compared to trials where the computer selected the play icon (t_39_ = 5.09, p < 0.001; see Figure 2 and Table S2). The personal control manipulation also affected the hedonic response to winning outcomes: “pleased with result” ratings were significantly higher on participant-chosen wins compared to computer-chosen wins (t_39_ = 2.50, p = 0.017; see Table S2).

The subjective response to the near-miss outcomes was striking: near-misses were experienced as aversive according to the ratings of “pleased with result,” but simultaneously increased ratings of “continue to play.” Both effects varied as a function of personal control (“pleased with result” control × outcome interaction F_2,78_ = 12.3, p = 0.001; “continue to play” control × outcome interaction F_2,78_ = 6.50, p = 0.002). Compared to computer-chosen near-misses, participant-chosen near-misses were significantly less pleasant (t_39_ = −4.21, p < 0.001) but significantly more motivating (t_39_ = 4.69, p < 0.001). Compared to participant-chosen full-misses, participant-chosen near-misses were significantly less pleasant (t_39_ = −2.75, p = 0.009) and significantly more motivating (t_39_ = 2.66, p = 0.011; see Figure 2). Further post hoc analysis revealed that the effect of the near-misses to increase ratings of “continue to play” on participant-chosen trials was predominantly driven by trials where, during reel spinning, the play icon moved through the payline and stopped in the next position (mean z = +0.18), compared to near-misses where the chosen icon stopped one position short of the payline (mean z = −0.19; t_39_ = 3.90, p < 0.001).

### Experiment II: Neural Correlates of the Slot Machine Task

**Figure 3:**
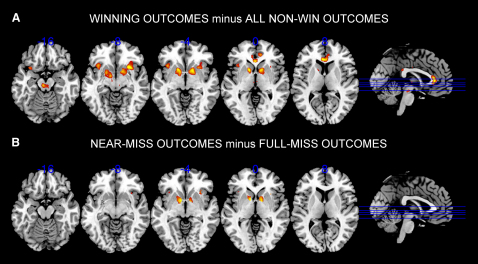
Neural Activity to Winning and Near-Miss Outcomes (A) Neural responses to monetary wins compared to all non-wins, modeled to the onset of the outcome phase. Suprathreshold voxels (p < 0.05 corrected for multiple comparisons) are displayed across 5 axial sections on the ch2bet template, using MRIcron software (http://www.sph.sc.edu/comd/rorden/mricron/). There was significant win-related activity in the ventral putamen (left: −14 10 −2; right 16 4 −12), anterior insula (left: −36 18 −4; right 28 16 −10), midbrain (−6 −20 −14) and rostral anterior cingulate cortex (−4 32 6) (see Table S3). (B) Neural responses to near-miss outcomes compared to full-miss outcomes, using a mask of win-related activity (mask thresholded at p_FWE-corr_ < 0.05). The contrast map has been thresholded at the lower level of p < 0.001 uncorrected to illustrate the anatomical extent of the clusters. Near-misses were associated with significant activity (p_FWE-corr_ < 0.05) in the bilateral ventral putamen (left: −8 4 −2; right 12 2 −2) and right anterior insula (32 18 0).

Brain responses during gambling play were measured using fMRI in a second group of 15 volunteers. Subjects played a longer version of the task comprising 3 blocks of 60 trials, yielding a total of 30 wins (£15), and subjective ratings were only taken intermittently (on 1 in 3 trials). An event-related analysis was used to identify neural responses at the receipt of the outcome, with the selection and anticipation phases entered as covariates in the design matrix (see Supplemental Data). The contrast of winning outcomes against all nonwin outcomes yielded significant signal change (p < 0.05 after correction for family wise error rate) in a distributed circuit comprising the ventral striatum bilaterally, the anterior insula bilaterally, the rostral anterior cingulate cortex (rACC), the thalamus and a midbrain cluster in proximity to the dopaminergic cells in the substantia nigra/ventral tegmental area (Bunzeck and Duzel, 2006; D'Ardenne et al., 2008; see Figure 3A and Table S4). This circuit is reliably activated in previous imaging studies using both unconditioned and conditioned reinforcers (Berns et al., 2001; Breiter et al., 2001; Delgado et al., 2000; Elliott et al., 2000; Knutson et al., 2003; McClure et al., 2003). The win-related circuit was implemented as a mask for the further fMRI contrasts that were orthogonal to this comparison.

**Figure 4:**
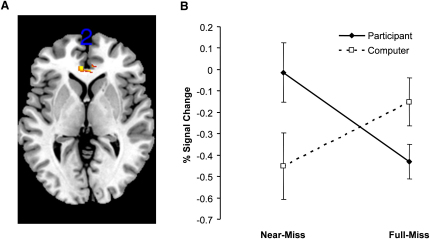
Near Miss by Control Interaction in Rostral ACC (A) The interactive effect of near-misses and personal control was associated with signal change in the rostral anterior cingulate cortex (rACC, Brodmann Area 24). The contrast image shown was restricted to near-miss outcomes that passed through the payline, compared to full-misses, using a mask of win-related activity (mask thresholded at p_FWE-corr_ < 0.05). For display purposes, the contrast map is thresholded at p < 0.001 uncorrected. (B) Extracted signal change from this cluster (averaged across all suprathreshold voxels using Marsbar) reveals that rACC activity was greater for near-misses (compared to full-misses) on participant-chosen trials (p = 0.005) but was lower for near-misses (compared to full-misses) on computer-chosen trials (p = 0.058). Error bars indicate standard error of the mean.

The contrast comparing the near-miss outcomes against the full-miss outcomes showed significantly elevated signal change to near-misses in the ventral striatum bilaterally (x, y, z = −8, 4, −2, Z = 4.30, p_FWE-corr_ = 0.005; 12, 2, −2, Z = 4.25, p_FWE-corr_ = 0.006) and the right anterior insula (x, y, z = 32, 18, 0, Z = 3.63, p_FWE-corr_ = 0.049), despite the lack of objective monetary gain on those trials (see Figure 3B). Signal increases in the ventral striatum and anterior insula to near-miss outcomes were present on trials with, and without, personal control. A third contrast assessed the interaction of the near-miss effect (near-misses minus full-misses) and personal control (participant-chosen trials minus computer-chosen trials) and identified bilateral voxels in the rACC region (Brodmann Area 24) with significance levels just below the familywise error-corrected threshold (x, y, z = 4, 32, 2, Z = 3.48, p_FWE-corr_ = 0.062; x, y, z = −4, 38, 2, Z = 3.36, p_FWE-corr_ = 0.088). When this contrast was confined to near-misses that had passed through the payline and stopped in the next position (given that the behavioral impact of near-misses was predominantly driven by this type), these voxels in the rACC were fully significant despite the restricted number of events (x, y, z = −4, 38, 2, Z = 4.34, p_FWE-corr_ = 0.005; x, y, z = 4, 34, 2, Z = 3.97, p_FWE-corr_ = 0.019; x, y, z = 6, 38, 2; z = 3.67, p_FWE-corr_ = 0.049). An analysis of extracted signal change from these voxels showed greater activity on near-misses compared to full-misses on participant-chosen trials (t_14_ = 3.37, p = 0.005), with a marginally significant effect in the opposite direction on computer-chosen trials (t_14_ = −2.06, p = 0.058; see Figure 4).

**Figure 5:**
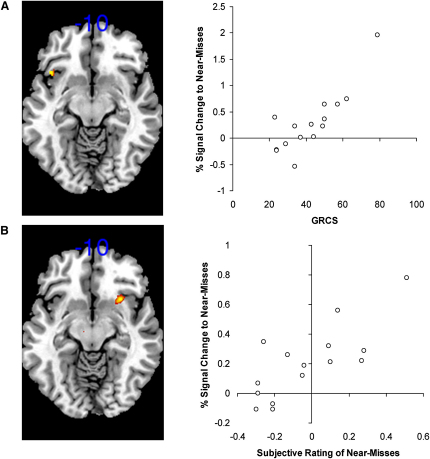
Insula Activation to Near-Misses Correlated with Trait Gambling Propensity and the Subjective Effect of Near-Misses (A) A trait measure of the susceptibility to gambling biases (the gambling-related cognitions scale; GRCS) predicted near-miss-related neural activity (contrast of near-misses minus full-misses) in the anterior insula/caudolateral orbitofrontal cortex (Brodmann Area 47). An SPM5 regression of near-miss-related activity onto GRCS total score, using a mask of win-related activity, revealed a single significant cluster in the left anterior insula (x, y, z = −42, 18, −10, z = 3.98, p_FWE-corr_ = 0.018), with extracted signal change displayed on the right hand side. For display purposes, the regression map is thresholded at p < 0.005 uncorrected. (B) Near-miss related activity in the anterior insula (Brodmann Area 13) was also significantly correlated with the subjective effects of near-miss outcomes on a rating of “How much do you want to continue to play the game?” The SPM5 regression map reveals a cluster in the right anterior insula (x, y, z = 30, 16, −10, z = 3.24, p_unc_ = 0.001), with extracted signal change displayed on the right hand side. For display purposes, the regression map is thresholded at p < 0.005 uncorrected.

Neural responses to the win and near-miss related contrasts were regressed against two sets of variables. First, we looked for brain areas where neural responses to wins and near-misses were correlated with the GRCS questionnaire, which assesses the susceptibility to cognitive distortions associated with gambling. GRCS total scores were well-distributed within the group (see Table S1). There were no significant voxels within the win-related circuit when GRCS score was regressed against win activity (wins minus all nonwins). However, when GRCS score was regressed onto near-miss activity (near-misses minus full-misses), there was a significant cluster in the anterior insula/caudolateral orbitofrontal cortex (BA 47; x, y, z = −42, 18, −10; z = 3.98, p_FWE-corr_ = 0.018; see Figure 5A). This correlation indicates that subjects who rated themselves as more susceptible to gambling distortions showed greater recruitment of the anterior insula in response to near-miss outcomes.

Second, we looked for brain areas where neural responses to wins and near-misses were correlated with the subjective ratings of “continue to play.” As ratings were acquired on one in three trials, we calculated a mean win effect for each subject (ratings on available win outcomes minus ratings on available nonwin outcomes) and a mean near-miss effect for each subject (ratings on available near-miss outcomes minus ratings on available full-miss outcomes). There were no significant voxels within the win mask when the subjective win effect was regressed against win-related brain activity. When the subjective near-miss effect was regressed against near-miss related brain activity, adjacent voxels in the anterior insula (Brodmann Area 13) and caudolateral orbitofrontal cortex (Brodmann Area 47) were the only significant effects at a reduced threshold of p < 0.001 uncorrected for multiple comparisons (x, y, z = 30, 16, −10, Z = 3.24, p_unc_ = 0.001; x, y, z = 34, 24, −4, Z = 3.37, p_unc_ < 0.0001). This correlation indicates that subjects who reported greater subjective effects of the near-misses on their ratings of “continue to play” showed a greater BOLD response to near-miss outcomes in the anterior insula/caudolateral orbitofrontal cortex (see Figure 5B).

## Discussion

The main focus of this study was the comparison of two types of nonwin outcome: near-misses, where the slot machine reel stopped one position from the chosen icon, and full-misses, where the outcome was not proximal to a win. While the objective outcome on these two trial types was the same (i.e., zero gain), there were significant differences between the patterns of neural response to the near-misses and full-misses. Near-misses were associated with significantly greater BOLD signal in the ventral striatum and anterior insula; areas that were also activated by unpredictable monetary wins on the task. This win contrast detected additional responses in the rACC, midbrain, and thalamus, confirming a well-established circuit of areas linked to reinforcement processing (Berns et al., 2001; Breiter et al., 2001; Elliott et al., 2000; Nieuwenhuis et al., 2005b; Thut et al., 1997), often referred to as the mesolimbic reward system (Elliott et al., 2000; Reuter et al., 2005). We propose that the recruitment of win-related regions during near-miss outcomes underlies their ability to promote gambling behavior. Previous studies have reported that moderate frequencies of near-misses (∼30%) encourage gambling persistence on slot machine simulations (Cote et al., 2003; Kassinove and Schare, 2001). By measuring the subjective response to outcomes on the slot machine task, we were able to better characterize these near-miss experiences. Although near-misses were rated as more unpleasant than full-misses, they simultaneously increased the desire to play the game. This invigorating effect depended upon a second factor, of personal control: near-misses only increased the desire to play when the subject had direct control over arranging their gamble. The interaction between near-misses and personal control was also evident in the fMRI data. In the rostral portion of the ACC, anterior to the genu of the corpus callosum, participant-chosen near-misses were associated with significantly greater BOLD signal than participant-chosen full-misses, whereas the opposite effect was observed on computer-chosen trials, albeit at a level that was not statistically reliable.

The anterior insula was recruited during both monetary wins and near-miss outcomes, and in addition, the BOLD response to near-misses in this region was associated with two sets of psychological variables. There was a significant positive correlation between insula activity to near-misses and the GRCS, a questionnaire measure of the susceptibility to gambling biases. We also observed a significant positive correlation between insula activity to near-misses and the subjective ratings of the near-misses on “How much do you want to continue to play the game?” In each case, the insula was the only area in the win-related circuit to show these predictive relationships. Thus, the neural response to near-miss outcomes in the anterior insula was associated with both the subjective impact of those events during scanning and a trait-related index of gambling propensity that is significantly elevated in problem gamblers (Raylu and Oei, 2004). While the significant insula foci were differentially lateralized (GRCS, left insula; subjective ratings, right insula), contralateral foci were apparent in both regressions when the statistical threshold was lowered (data not shown), and therefore we do not infer any meaningful lateralization from these results. These correlations lend support to the ecological validity of our task. Moreover, they suggest that the anterior insula may be a key locus in mediating the invigorating effects of near-miss outcomes on gambling behavior.

Our insula data are congruent with accumulating evidence for insula involvement in drug craving and extend these findings to gambling as a behavior that can become addictive (Potenza, 2006). Functional imaging studies in cocaine addicts have previously reported insula activation during exposure to cocaine-related stimuli (Garavan et al., 2000; Wang et al., 1999), which was correlated with subjective reports of the degree of induced craving (Wang et al., 1999). A neuropsychological study has reported that patients with insula damage showed rapid cessation of cigarette smoking without persistent urges to smoke, compared to brain-injured patients with damage to other regions (Naqvi et al., 2007). A study in experimental animals also showed that temporary inactivation of the insula reduced amphetamine seeking behavior in amphetamine-experienced rats (Contreras et al., 2007). Given its well-recognized role in the processing of bodily feedback (Craig, 2002), the insula's involvement in addictive behaviors may be to signal the interoceptive aspects of compulsive urges (Gray and Critchley, 2007). While previous fMRI studies have often associated insula activity with negative emotional states like disgust (Phillips et al., 1997), pain (Ploghaus et al., 1999), or risk of financial loss (Paulus et al., 2003), it is also reliably recruited in response to monetary gains (Delgado et al., 2000; Elliott et al., 2000; Izuma et al., 2008) and other appetitive processing (Craig, 2002). Based on the present findings, we would hypothesize that excessive insula recruitment during gambling play may be a risk factor for the cognitive distortions and loss-chasing that are characteristic of problem (“compulsive”) gambling.

These findings provide a number of further insights into the psychological basis of the near-miss effect. By one account, the near-miss may be conceptualized as the omission of an expected reward and considered within the context of fMRI studies of reinforcement learning by prediction error (e.g., D'Ardenne et al., 2008; McClure et al., 2003; O'Doherty et al., 2003). By this formulation, on near-miss trials, as the slot machine reel approaches a standstill during the anticipation phase, the subject develops an expectation that they are about to win. This may be analogous to an effect shown in electrophysiological research, that during appetitive Pavlovian conditioning tasks with uncertain rewards, there is a “ramping up” of mesolimbic dopamine cell firing between the CS presentation and the expected time of juice delivery (Fiorillo et al., 2003). In our slot machine task, this positive prediction error is rapidly followed by a negative prediction error in the outcome phase, as the expected win is withheld. Dopamine cells show a pause in firing to omission of an expected reward (Schultz, 2006). The observed signal in the ventral striatum during near-miss outcomes is compatible with this account, as BOLD response in this region is known to correlate closely with both positive and negative prediction errors (D'Ardenne et al., 2008; McClure et al., 2003; O'Doherty et al., 2003) and is observed irrespective of instrumental demands (O'Doherty et al., 2004; Zink et al., 2003), consistent with our finding that this region responded to near-misses across both participant-chosen and computer-chosen conditions. However, several aspects of this formulation remain unclear: how positive and negative prediction errors may summate when occurring close together in time, how pauses in dopamine cell firing influence the BOLD response, and whether reward omission and discrete “nonwin” events are analogous. Future research may fruitfully manipulate the expectation of winning in order to study its effect upon near-miss processing.

Several aspects of the current data present problems for an account of the near-miss effect solely in terms of reward expectation and omission. First, why should unpleasant reward omission invigorate behavior? It is unclear how this account would explain our finding that near-misses increased self-report ratings of desire to play the game. In many real-world situations (e.g., target practice), near-miss outcomes are indicative of skill acquisition and, as such, constitute useful signals of imminent success. In these environments, it is advantageous for reinforcement learning algorithms to compute a value function that can assign some value to near-misses, despite the objective absence of reinforcement on these trials (e.g., Daw et al., 2006; Kakade and Dayan, 2002). In many gambling games, however, winning outcomes are chance events and near-misses are not predictive of winning, and so it would be misleading to assign value to near-misses. Humans are often deficient at processing chance events (Carlson and Shu, 2007; Wagenaar, 1988), and it is conceivable that gambling games may harness a reinforcement learning system that evolved to handle skill-oriented behaviors.

A second issue is that the reward expectation and omission components were matched across the participant-chosen and computer-chosen conditions, but we saw differences between the near-miss outcomes as a function of personal control, in both the subjective ratings and the rACC response. These effects are more consistent with cognitive formulations of gambling that invoke an “illusion of control,” where the gambler interprets the near-miss as evidence that he has acquired skill at the game. Crucially, this appraisal of skill is most likely to occur when the player has control over their gamble selection. Thus, by interpreting the near-miss event as evidence of skill acquisition, the subject is motivated to continue gambling in order to exploit this (perceived) knowledge. Our finding that the rACC is sensitive to this manipulation is consistent with much that is known about ACC involvement in reward processing and decision-making. Lesion studies in experimental animals have shown a critical role for ACC in deciding how much effort to invest in obtaining a reward (Rudebeck et al., 2006) and in using the outcomes of past decisions to guide ongoing choice (Behrens et al., 2007; Kennerley et al., 2006). The dependency of the rACC response upon personal control is also consistent with event-related potential data where the feedback negativity (thought to derive from a medial frontal source) was enhanced on trials where a choice or response was required (Yeung et al., 2005; see also Walton et al., 2004 for similar data using fMRI). In the specific context of gambling behavior, we postulate that the rACC plays a key role in processing the personal significance of the near-miss outcomes; that is, interpreting them as evidence of skill acquisition and using these outcomes to inform subsequent choice.

An alternative, lower-level account of these near-miss phenomena is that they are an effect of perceptual generalization. By virtue of their spatial proximity to the goal state, near-misses may engender some goal-related neural processing. Such an account seems unlikely for two reasons. First, spatial proximity to the payline is equally present across the participant-chosen and computer-chosen conditions, but the impact of the near-misses on ratings of “continue to play” and rACC signal diverged as a function of personal control. Second, both the subjective ratings and the rACC signal were predominantly modulated by near-misses that passed through the payline before stopping; the near-misses that stopped one position short of the payline were much less effective. A perceptual generalization account would predict comparable effects for near-misses either side of the payline.

The effects of near-misses and personal control in the present study were observed in healthy volunteers who did not gamble regularly. When nine subjects with moderate gambling involvement (South Oaks Gambling Screen scores 2–5) were excluded from the behavioral analysis (n = 31), the potency of the near-miss and personal control manipulations were unaffected (see Supplemental Data). However, as gambling becomes dysfunctional and problematic, it is likely that these cognitive distortions become exacerbated (Joukhador et al., 2003), along with disruption of multiple components of reward-related brain circuitry. A previous fMRI study in pathological gamblers using a card-guessing task reported blunted activation in the ventral striatum and ventromedial prefrontal cortex (which includes the rACC) in a contrast of monetary wins and losses (Reuter et al., 2005). Similar findings were reported in substance addictions (Goldstein et al., 2007; Wrase et al., 2007). Conversely, human lesion patients with damage to the ventromedial prefrontal cortex showed increased wagering on a neuropsychological test of gambling behavior (Clark et al., 2008). Activity in this region has also been associated with decisions to chase one's losses (Campbell-Meiklejohn et al., 2008), which is widely recognized as a hallmark of problem gambling. As well as the near-miss effects discussed above, the presence of personal control caused subjects to rate their chances of winning as higher, and the winning outcomes as more pleasurable, compared to computer-selected gambles, although we were unable to detect any corollary of these effects in the fMRI experiment.

These data demonstrate that two cognitive distortions associated with gambling behavior can be elicited in a laboratory setting, in healthy subjects who do not gamble with any regularity. Gambling near-misses were associated with significant recruitment of brain win-related circuitry and acted to increase desire to gamble when the subject had personal control over selecting the gamble. These neural responses may be described as anomalous, in the sense that they occur in the absence of objective reinforcement on near-miss trials. In this sense, these findings are congruent with data showing that striatal activity is tied to the subjective utility rather than the objective value of the outcome; for example, the ventral striatal response to monetary wins is also sensitive to framing effects (Nieuwenhuis et al., 2005a) and individual discount functions in the valuation of delayed rewards (Kable and Glimcher, 2007). These neural correlates of the near-miss effect may underlie the behavioral potency of near-miss outcomes to engender continued play. Our findings in the ventral striatum and rACC are consistent with current knowledge about the involvement of these regions in reward processing and decision-making. The close relationships between insula recruitment, and measures of gambling propensity and the subjective effects of near-misses, indicate an important role for the insula in decisions to gamble. By linking psychological and neurobiological accounts of gambling behavior, these data inform our understanding of the allure of gambling behavior within society, and by extrapolation, the capacity of gambling to become addictive and pathological.

## Experimental Procedures

### Experiment 1 (Behavioral Study)

#### Subjects

Forty undergraduate volunteers (23 male) were recruited through university advertisements that asked “Do you enjoy gambling?” Subjects attended a single test session, where they completed the slot machine task (30 min), the South Oaks Gambling Screen (Lesieur and Blume, 1987) and the gambling-related cognitions scale (Raylu and Oei, 2004; see Table S1). The protocol was approved by the Cambridge Psychology Research Ethics Committee (#2006.35) and all volunteers provided written informed consent. Volunteers were instructed that they would have “the opportunity to win money on the task” and by virtue of the pseudorandomized win sequence, all participants received £5 at the end of the session.

#### Task Design

The task was programmed in Microsoft Visual Basic 6, with responses registered on three adjacent keyboard keys. Trial structure and display screen are displayed in Figure 1. The task display resembles a two-reel slot machine, with the same six icons displayed, in the same order, on the left and right reel, and a horizontal “payline” across the center of the screen. At the start of the task, the subject was invited to select the six icons they wished to play with, from sixteen alternatives arranged in a 4 × 4 matrix. This feature was included to enhance the participants' level of involvement, and subjects were instructed that the available shapes would vary in the chances of winning during the game. After selecting their icons, the subject played 4 practice trials followed by 60 trials with monetary reward available.

Each trial consisted of a *selection* phase, an *anticipation* phase, and an *outcome* phase. The selection phase lasted a fixed 5 s duration, where one shape on the left reel was selected. The anticipation phase lasted a variable duration (2.8–6 s), where the right reel was spun, and decelerated to a standstill. The outcome phase was initiated as the right reel stopped moving: if the right reel stopped on the icon that was selected on the left reel (i.e., matching icons displayed in the payline), the subject was awarded £0.50; all other outcomes won nothing. Outcomes were presented in a fully balanced pseudorandom order to ensure a proportionate number of wins over the 60 trials (1/6, total 10), near-misses (2/6, total 20) and full-misses (3/6, total 30). The outcome phase lasted a fixed 4 s duration. At the end of each trial, there was an intertrial interval of variable duration (2–7 s).

Two trial types were presented in a pseudorandom order: on 30 trials, the play icon was selected by the participant, and on the other 30 trials, the play icon was selected by the computer. Trial type was indicated by screen background color and a message on the left side of the screen. On participant-chosen trials (white background), the subject chose the play icon using keys 1 and 2 to rotate the reel up and down, and key 3 to select the icon currently displayed in the payline. On computer-chosen trials (black background), the computer selected the icon on the left reel, but the subject was required to confirm the selection by pressing key 3, in order to better equate attentional and motor demands across the two conditions. In both conditions, if selection/confirmation was not completed within the 5 s window, a “Too late” message was displayed and the next trial commenced after the intertrial interval.

On each trial, subjective ratings were also acquired using onscreen visual analog scales. After the selection phase, subjects rated “How do you rate your chances of winning?” and after the outcome phase, two further ratings were taken: “How pleased are you with the result?” and “How much do you want to continue to play the game?” Subjects indicated their response on a 21 point scale using keys 1 and 2 to move left and right and key 3 to confirm. No time limit was imposed for the subjective ratings.

#### Statistical Analysis

Subjective ratings for each subject were standardized to a z score, based on the individual's mean and standard deviation for that rating, given the variability in anchoring across subjects. Data were inspected for normality and parametric statistics were applied only where normality was met. Subjective ratings were analyzed using paired t tests (for “chances of winning”) and repeated-measures analysis of variance (for “pleased with outcome” and “continue to play”) with outcome (three levels: win, near-miss, full-miss) and control (two levels: participant-chosen, computer-chosen) as factors. The Greenhouse-Geisser correction was applied where sphericity was violated. Two-tailed statistics were thresholded at p < 0.05.

### Experiment 2 (fMRI Study)

#### Subjects

Seventeen right-handed subjects with no history of psychiatric or neurological disorder were recruited from advertisements around the University. One subject withdrew due to claustrophobia, and one subject was subsequently excluded from all analysis due to excessive movement (∼4 mm within session, with pronounced spiking), leaving 15 subjects (9 male, mean age 26 SD 7.5) in the reported analysis. Subjects reported minimal to modest involvement in gambling, indexed by scores on the South Oaks Gambling Screen of 0–3 (mean 0.7, SD 1.0; scores ≥5 indicate probable pathological gambling). Subjects attended a single fMRI session at the Wolfson Brain Imaging Centre in Cambridge, UK. The protocol was approved by the Norfolk & Norwich Research Ethics Committee (COREC 06/Q0101/69) and all volunteers provided written informed consent. Volunteers were reimbursed £20 for participation “with the opportunity to win further money on the task” (£15 over 180 trials).

#### Task Design

Several minor modifications were required for fMRI. First, the auditory feedback was not delivered given the noise of the scanner. Second, more trials were acquired (3 blocks of 60 trials) to enable sufficient power for fMRI analysis. Third, the number of ratings was reduced given the longer task duration: the “pleased with outcome” rating was dropped altogether, and the ratings of “chances of winning” and “continue to play” were acquired, at random, on 1 in 3 trials. Responses were recorded using the first three buttons on a four-button box, resting on the subject's stomach under the dominant hand. Subjects performed 10 practice trials on the task (delivering two hypothetical wins) before entering the fMRI scanner.

#### Imaging Procedure

Scanning was performed on a Siemens TimTrio 3 Tesla magnet using a 32 slice axial oblique sequence, with a repetition time of 2 s (TE 30ms, flip angle 78°, voxel size 3.1 × 3.1 × 3.0 mm, matrix size 64 × 64, field of view 201 mm × 201 mm, bandwidth 2232 Hz/Px). At the start of each run, six dummy scans were discarded to allow for equilibrium effects. Each 60 trial EPI run lasted a maximum of 630 repetitions (21 min), but was terminated early on block completion. A high-resolution T1-weighted three-dimensional magnetization-prepared rapid acquisition gradient-echo sequence (MP-RAGE) structural image was also acquired for use in spatial normalization of the EPI series.

#### Imaging Analysis

fMRI data analysis was performed using SPM5 (Statistical Parametric Mapping, Wellcome Department of Cognitive Neurology, London, UK). Data preprocessing consisted of slice timing correction, within-subject realignment, spatial normalization, and spatial smoothing using a 10 mm Gaussian kernel. Time series were high pass filtered (128 s). Volumes were normalized to the International Consortium for Brain Mapping (ICBM) templates that approximate to Talairach and Tournoux (1988) space, using a matrix obtained from normalizing each subject's segmented MP-RAGE structural scan onto the ICBM gray and white matter templates.

A canonical hemodynamic response function (HRF) was modeled to the onsets of the selection phase, the anticipation phase and the outcome phase on each trial. At the selection onset, two trial types were distinguished: participant-chosen trials and computer-chosen trials. At anticipation and outcome, eight trial types were distinguished, comprising a 2 (choice: participant-chosen, computer-chosen) by 4 (win, near-miss before the payline, near-miss past the payline, full-miss) factorial design. The design matrix thus comprised 18 (2 + 8 + 8) columns per session. In addition, the movement parameters from realignment were included as covariates of no interest. The HRF was used a covariate in a general linear model, and a parameter estimate was obtained for each voxel, for each event type, reflecting the strength of covariance between the data and the canonical HRF. Contrast images were calculated between parameter estimates from different trial types. The following outcome-related contrasts were computed:

(1) Win-related activity: Winning outcomes on participant- and computer- chosen trials minus all nonwinning outcomes on participant- and computer-chosen trials.
(2) Near-miss activity: near-miss outcomes on participant- and computer-chosen trials minus full-miss outcomes on participant- and computer-chosen trials. This contrast was restricted to areas showing win-related activity (i.e., masked with contrast 1).
(3) Near-miss by choice interaction: areas differentially recruited by near-misses compared to full-misses as a factor of participant versus computer control (i.e., 1, −1, −1, 1). This contrast was restricted to areas showing win-related activity (i.e., masked with contrast 1).
(4) Win activity as a function of personal control: winning outcomes on participant-chosen trials minus winning outcomes on computer-chosen trials. This contrast was restricted to areas showing win-related activity (i.e., masked with contrast 1).

Based on the data from experiment 1, contrasts 2 and 3 were repeated restricted to near-miss outcomes where the play icon passed through the payline. Contrasts 1 and 2 were also calculated for the onset of the anticipation phase. For the selection phase onsets, a single contrast of participant-chosen minus computer-chosen trials was calculated (see Table S5). Individual contrast images were taken to a second-level random-effects group analysis, and were thresholded at p < 0.05 corrected for multiple comparisons using random field theory (Worsley et al., 1996), i.e., familywise error corrected. Masking of contrasts with the win-related activity (contrast 1) was performed using the PickAtlas tool (Maldjian et al., 2003). Signal change was extracted from activated foci using the MARSBAR tool (http://marsbar.sourceforge.net/) for the purposes of plotting the data.

## Supplemental Data

Document S1. Five Tables and Supplemental Text
