# Supplementary material for: Gambling Near-Misses Enhance Motivation to Gamble and Recruit Win-Related Brain Circuitry
Source: Neuron. 2009 Feb 12;61(3):481–90. doi: 10.1016/j.neuron.2008.12.031 (PMC2658737; doi:10.1016/j.neuron.2008.12.031)
Supplement: Document S1. Five Tables and Supplemental Text [file mmc1.pdf]

Neuron, Volume *61*

Supplemental Data

Gambling Near-Misses Enhance Motivation to Gamble and Recruit Win-Related Brain Circuitry

Luke Clark, Andrew J. Lawrence, Frances Astley-Jones, Nicola Gray

Behavioral Study (Experiment 1)

Supplementary Results

The number of response omissions during the selection phase was low overall (mean over 60 trials =2.33 sd 2.35) but omissions were more likely on participant-chosen trials (mean 1.45) than computer-chosen trials (mean 0.88) (Wilcoxon signed ranks Z=2.51, p=.012).

The 3 (outcome) x 2 (control) repeated-measures ANOVA of 'pleased with result' showed significant main effects of outcome ($F_{2,78}$=333.6, p<.001 Greenhouse-Geisser ε=.557) and control ($F_{1,39}$=4.49, p=.040), and a significant outcome x control interaction ($F_{2,78}$=12.3, p<.001 Greenhouse-Geisser ε=.769). Analysis of simple main effects looked at the effect of agency at each level of outcome. On winning outcomes, ratings were higher on participant-chosen trials than computer-chosen trials (t_{39}=2.50, p=.017). On near-miss outcomes, ratings were significantly lower on participant-chosen trials than computer-chosen trials (t_{39}=-4.21, p<.0001). On full-miss outcomes, ratings were significantly lower on participant-chosen trials than computer-chosen trials (t_{39}=-3.21, p=.003). Participant-chosen near-misses were significantly less pleasant than participant-chosen full-misses (t_{39}=-2.75, p=.009), and there was no difference between near-misses and full-misses on computer-chosen trials (t_{39}=.721, p=.475).

The 3 (outcome) x 2 (control) repeated-measures ANOVA of 'continue to play' showed significant main effects of outcome ($F_{2,78}$=67.1, p<.001, Greenhouse-Geisser ε=.690) and control

($F_{1,39}$=6.40, p=.016), and a significant outcome x control interaction ($F_{2,78}$=6.50, p=.002). Analysis of simple main effects looked at the effect of agency at each level of outcome. On near-miss outcomes, ratings were significantly higher on participant-chosen trials than computer-chosen trials (t_{39}=4.69, p<.0001). There were no significant differences as a function of agency on wins (t_{39}=.931, p=.358) or full-misses (t_{39}=1.49, p=.144). On participant-chosen trials, near-misses were rated significantly higher than full-misses (t_{39}=2.66, p=.011). On computer-chosen trials, near-misses were rated significantly lower than full-misses (t_{39}=-3.09, p=.004).

A further analysis looked for differential effects of near-miss position, i.e. whether the right-hand reel moved through the payline and stopped in the next position ('type I') or stopped one position short of the payline ('type II'). A 3 (outcome: near miss type I, near miss type II, full-miss) x 2 (control) ANOVA on ratings on 'continue to play' revealed a significant outcome x control interaction (F 2,78=24.5, p<.001), with a highly significant difference between participant-chosen and computer-chosen near-misses on type I trials (t_{39}=7.46, p<.001) but no difference on type II trials (t_{39}=1.00, p=.323). The equivalent ANOVA for ratings of 'pleased with result' did not indicate differential effects of the two near-miss types ($F_{2,78}$=1.11, p=.336).

Finally, we conducted two sets of analyses to explore the relationship between the subjective ratings on the slot machine task, and level of gambling involvement (see Supplementary Table 1). First, scores on the South Oaks Gambling Screen varied from 0-5, with 31 subjects scoring 0-1. The basic analyses of the three sets of ratings were repeated in this restricted group of non-gamblers (i.e. excluding those subjects with SOGS scores ≥2), and the statistical findings were unchanged. Thus, the efficacy of the personal control manipulation and the near-miss effect cannot be attributed to a minority of subjects with moderate gambling involvement. Second, we examined correlations between the GRCS (see Supplementary Table 1) and several parameters from the subjective ratings: 1) the effect of personal control ('chances of winning' on participant-

chosen trials minus computer-chosen trials), 2) the effect of winning outcomes on ‘pleased with result’ (wins minus all non-win outcomes), 3) the effect of winning outcomes on ‘continue to play’ (wins minus all non-win outcomes), 4) the effect of near-misses on ‘continue to play’ (participant-chosen near-misses minus computer-chosen near-misses). Higher scores on the GRCS were associated with a greater effect of winning outcomes on ratings of ‘continue to play’ (r_{40}=.328, p=.039), supporting the ecological validity of the task. There were no further significant correlations (all r<.12).

Experiment 2 (fMRI study)

Behavioral Data

The number of response omissions during the selection phase was low overall (mean over 180 trials =2.40 sd 1.50) and the number of omissions did not differ between participant-chosen trials (mean 1.00) and computer-chosen trials (mean 1.40) (Wilcoxon signed ranks Z=.782, p=.434), indicating comparable attention across the two conditions.

Subjective ratings of “How do you rate your chances of winning?” were compared across participant-chosen and computer-chosen trials using a paired t test. As in Experiment 1, subjects rated their chances of winning as significantly higher on participant-chosen trials (t_{14}=5.78, p<.001). Subjective ratings of “How much do you want to continue to play the game?” were analysed using a 3 (outcome) x 2 (control) repeated-measures ANOVA, which yielded a significant main effect of outcome ($F_{2,28}$=15.8, p<.001, Greenhouse-Geisser Epsilon=.666) and a significant outcome x control interaction ($F_{2,28}$=8.19, p=.002). Simple main effects analysis indicated that participant-selected wins increased ratings more than computer-selected wins (t_{14}=3.72, p=.002). There were no significant differences between near-misses and full-misses in the participant-chosen trials (t_{14}=1.52, p=.150) or the computer-chosen trials (t_{14}=1.38, p=.190). However, inspection of mean ratings (see Supplementary Table 3) indicated the same trend as the

behavioral experiment. When the participant-chosen comparison was restricted to near-misses that had passed through the payline, the near-misses were rated as significantly more motivating (mean z=+.01) than full-misses (mean z=-0.19) (t_{14}=2.26, p=.040).

To summarise the ratings data, the manipulation of personal control was effective in the fMRI procedure, as evidenced by i) increased ratings of 'chances of winning' on participant-chosen trials, and ii) increased ratings of 'continue to play' after a win on participant-chosen trials. The near-miss effect was evident in a significant outcome x control interaction on ratings of 'continue to play', and in a paired t test comparing near-misses below the payline against full-misses. The attenuated effect sizes relative to the behavioral experiment are likely attributable to the smaller number of subjects and the intermittent nature of the ratings (1 in 3 trials).

FMRI Data

Contrast 1 (Win-related activity at outcome): see Supplementary Table 4.

Contrast 2 (Near-miss related activity at outcome): Using contrast 1 as a mask, the contrast of near-misses minus full-misses yielded significant activation in 3 clusters; left ventral striatum (x, y, z=-8, 4, -2, z=4.30, $p_{FWE\text{-}corr}$=.005), right ventral striatum (12, 2, -2, z=4.25, $p_{FWE\text{-}corr}$ =.006) and right anterior insula (x, y, z=32, 18, 0, z=3.63, $p_{FWE\text{-}corr}$ =.049). When this analysis was restricted to near-misses that had passed through the payline (type I near-misses), there were no supra-threshold voxels.

Contrast 3 (Near-miss X Control interaction at outcome): Using contrast 1 as a mask, the interaction map for the near-miss effect as a function of personal control yielded no significant activations at $p_{FWE\text{-}corr}$<.05, although there were two clusters in rostral anterior cingulate cortex that approached significance (4, 34, 2, z=3.48, $p_{FWE\text{-}corr}$ =.062; -4, 38, 2, z=3.36, $p_{FWE\text{-}corr}$ =.088). When this analysis was restricted to near-misses that had passed through the payline (type I near-

misses), there were three significant clusters in the rostral anterior cingulate cortex: -4, 38, 2 ($z=4.34$, $p_{FWE\text{-}corr}=.005$), 4, 34, 2 ($z=3.97$, $p_{FWE\text{-}corr}=.019$) and 6, 38, 2 ($z=3.67$, $p_{FWE\text{-}corr}=.049$).

Contrast 4 (Wins as a function of Control, at outcome): no supra-threshold voxels.

Contrast 5 (Selection phase activity): see Supplementary Table 5. Icon selection on participant-chosen trials, compared to computer-chosen trials, was associated with significant activation across a distributed network comprising cerebellum, bilateral parietal cortex, bilateral premotor cortex, thalamus, striatum and cingulate gyrus.

Contrast 6 (Win-related activity during anticipation phase): no significant voxels.

Contrast 7 (Near-miss related activity during anticipation phase): no significant voxels.

Supplementary Table 1: Gambling self-report data from the South Oaks Gambling Screen and Gambling-Related Cognitions Scale (GRCS) in Experiments 1 and 2 (Mean (sd)).

	Experiment 1	Experiment 2
n	40	15
South Oaks Gambling Screen	0.9 (1.13) (range 0-5)	0.67 (0.98) (range 0-3)
GRCS-Total	51.0 (19.6)	42.6 (15.8)
GRCS Subscales:*		
Gambling Expectancies	9.48 (3.49)	7.67 (4.02)
Illusion of Control	7.95 (5.02)	6.87 (3.31)
Predictive Control	14.9 (7.00)	12.5 (4.30)
Inability to Stop	6.6 (4.98)	5.87 (2.59)
Interpretive Bias	12.1 (5.65)	9.73 5.56)

* The GRCS is a 23-item self-report questionnaire with each item rated on a scale from 1 (strongly disagree) to 7 (strongly agree). Example items on GRCS:

Gambling Expectancies (#1): “Gambling makes things seem better”

Illusion of Control (#8): “Specific numbers and colours can help increase my chances of winning”

Predictive Control (#4): “Losses when gambling are bound to be followed by a series of wins”

Inability to Stop (#17): “I’m not strong enough to stop gambling”

Interpretive Bias (#10): “Relating my losses to bad luck and bad circumstances makes me continue gambling”

Supplementary Table 2: Subjective ratings from behavioral validation study (Experiment 1; n=40)

		Participant-Chosen	Computer-Chosen
"How do you rate your chances of winning?"(0=very low, 100=very high)			
	Raw	35.1 (14.8)	29.9 (12.7)
	Z	0.16 (0.20)	-0.16 (0.20)
"How pleased are you with the result?" (-100=very unhappy, 0=neutral, +100=very happy)			
Wins	Raw	41.2 (31.8)	35.7 (29.2)
	Z	1.67 (0.60)	1.48 (0.56)
Near-misses	Raw	-34.0 (22.6)	-25.1 (21.9)
	Z	-0.47 (0.23)	-0.24 (0.22)
Full-misses	Raw	-29.8 (21.7)	-24.7 (21.5)
	Z	-0.36 (0.16)	-0.21 (0.23)
"How much do you want to continue to play?" (0=not at all, +100=a lot)			
Wins	Raw	52.6 (18.7)	52.5 (15.9)
	Z	0.59 (0.50)	0.52 (0.40)
Near-misses	Raw	45.7 (14.3)	42.4 (15.6)
	Z	0.0 (0.25)	-0.23 (0.19)
Full-misses	Raw	43.2 (15.4)	44.7 (13.5)
	Z	-0.15 (0.19)	-0.08 (0.22)

Supplementary Table 3: Subjective ratings from fMRI study (Experiment 2; n=15)

		Participant-Chosen	Computer-Chosen
“How do you rate your chances of winning?” (0=very low, 100=very high)			
	Raw	51.3 (16.7)	43.9 (14.6)
	Z	0.23 (0.15)	-0.23 (0.16)
“How much do you want to continue to play?” (0=not at all, +100=a lot)			
Wins	Raw	80.0 (13.1)	75.5 (12.5)
	Z	0.64 (0.37)	0.28 (0.52)
Near-misses	Raw	69.7 (14.3)	68.5 (13.0)
	Z	-0.09 (0.25)	-0.13 (0.25)
Full-misses	Raw	67.9 (14.3)	71.6 (11.8)
	Z	-0.19 (0.22)	0.03 (0.29)

Supplementary Table 4: Group activation foci in the Win-related contrast (all winning outcomes minus all non-win outcomes)

		Talairach coordinates				
Location	Side	x	y	z	Z	$P_{FWE\text{-}corr}$
Caudolateral OFC / Insula Cortex (BA 47)	R	28	16	-10	6.21	<.001
	L	-36	18	-4	5.59	.002
Ventral Striatum	R	16	4	-12	6.02	<.001
	R	16	6	-4	5.82	<.001
	L	-14	10	-2	5.91	<.001
Globus Pallidus	L	-10	-2	-6	5.60	.002
Anterior Cingulate (BA 24)	L	-4	32	6	6.10	<.001
	R	6	28	20	5.07	.028
BA 32	R	10	40	2	5.85	<.001
Midbrain	L	-6	-20	-14	5.72	.001
	R	8	-20	-12	5.53	.003
	L	-8	-12	-8	5.07	.028
Posterior Cingulate	L	-2	-34	28	5.25	.013
	R	2	-14	32	5.18	.018
	R	4	-4	34	5.09	.026
	R	4	-20	32	5.03	.033
Cerebellum	R	30	-66	-26	5.48	.004
Thalamus	R	2	-16	2	5.18	.018

Supplementary Table 5: Group activation foci in the Selection phase contrast (participant-chosen trials minus computer-chosen trials)

Location	Side	Talairach coordinates			Z	$P_{FWE\text{-}corr}$
		x	y	z		
Cerebellum	R	18	-44	-24	6.36	<.001
	R	8	-60	-40	5.66	.001
	R	4	-62	-14	5.15	.022
	L	-26	-50	-26	5.08	.031
Parietal Cortex	L	-34	-32	52	6.10	<.001
	L	-30	-46	60	5.11	.027
	L	-48	-32	40	5.41	.005
	R	48	-30	54	5.46	.004
Premotor Cortex (BA 6)	L	-22	-4	58	5.85	<.001
	L	-4	-4	54	6.00	<.001
	R	38	-10	52	4.96	.049
Thalamus	L	-12	-18	4	6.05	<.001
Midbrain	R	10	-12	-2	5.82	<.001
Cingulate Gyrus (BA 32)	R	6	22	30	5.91	<.001
Insula / Claustrum	R	32	14	2	5.55	.002
	L	-32	16	4	5.53	.003
Insula (BA 13)	L	-38	0	6	5.35	.007
	R	44	10	-4	5.04	.036
Putamen	L	-18	2	12	5.49	.003

	R	18	12	0	5.26	.012
Inferior Frontal Gyrus	L	-56	6	30	5.06	.033
Globus Pallidus	R	24	-10	2	4.96	.049
